# A dismantling study on imaginal retraining in overweight or obese women

**DOI:** 10.1038/s41398-021-01595-1

**Published:** 2021-09-17

**Authors:** Janina Wirtz, Leonie Ascone, Josefine Gehlenborg, Steffen Moritz, Simone Kühn

**Affiliations:** 1 Department of Psychiatry and Psychotherapy, University Medical Center Hamburg-Eppendorf (UKE), Hamburg, Germany; 2 Lise Meitner Group for Environmental Neuroscience, Max Planck Institute for Human Development, Berlin, Germany

**Keywords:** Human behaviour, Addiction

## Abstract

Imaginal retraining is a variant of approach bias modification and transfers the method into one’s own mind. As the technique contains multiple elements, this pilot study aimed to dismantle which of its components is most efficient in reducing craving for high-calorie food. A total of 113 women were randomly allocated to one out of six conditions containing a short intervention to mentally manipulate a picture displaying high-calorie foods. Four of the interventions involved different combinations of elements of the imaginal retraining technique, while the remaining two conditions comprised thought suppression or merely observing a picture. Participants rated their level of craving, as well as three pictures containing healthy and unhealthy foods regarding their pleasantness before and after the interventions took place. Within-group changes were assessed with paired *t*-tests (in case of non-normal data Wilcoxon paired *t*-tests) and between-group differences with one-way ANOVAs (non-parametric Kruskal–Wallis tests). A trend level reduction in craving was found in the imaginal retraining condition with and without a movement. A post hoc analysis of both conditions joint together showed a statistically significant reduction in craving. In addition, positive picture appraisal for unhealthy foods was significantly reduced in both imaginal retraining conditions (with and without movement) with medium to large effect sizes. This study demonstrated that imaginal retraining with an arm movement can reduce craving and picture appraisal for high-calorie foods significantly in a one-time application. It is a promising technique to reduce appraisal for unhealthy high-calorie foods. Future studies should repeat the experiment in situations of high craving and allow for a personalized selection of stimuli.

## Introduction

Obesity poses an immense risk on health and is the origin of a wide variety of diseases [1]. According to the World Health Organization [2], obesity is the result of an excessive increase in weight caused by an imbalance between consumed and expended calories. This is usually driven by increased intake of high-calorie foods, accompanied by physical inactivity [2]. Research has shown that eating behaviour in obese individuals displays neurobiological and phenomenological similarities to addictive disorders [3–5]. Craving plays a significant role in addictive behaviours and can be conceptualized as a psychological or physiological motivational state that advocates the consumption of a desired substance [6, 7]. Because of its intensity and specificity (e.g., directed at a certain substance or food), it can be distinguished from the regular perception of hunger [7, 8]. Craving occurs even when satisfied, can pass with time, can decrease in intensity, and is usually directed at high-calorie (sweet or savoury) ‘comfort’ foods. What type of food is craved can depend on a number of physical and mental factors ranging from nutritional imbalance, learned/conditioned behaviour, acquired habit and being subjected to food cues in the environment [8].

Research has shown that ‘thought suppression’ is effective in reducing craving short-term, however, a behavioural rebound effect is to be expected [9]. Similarly, thinking about the negative consequences of unhealthy food consumption rendered effective in reducing craving for unhealthy foods [10]. In particular, a study on mind-set training [11] showed that a ‘LATER mind-set’, which involves thinking about the long-term consequences of unhealthy food consumption, was successful in significantly reducing craving after a single training session. However, the longer-term effects of these mind sets have not been evaluated in the scope of the study. Regulation of craving training (ROC-T), a brief yet computerized training, has also been shown to reduce craving [12]. As the occurrence of craving is unpredictable, it is of high interest to develop, refine and enhance interventions that are quick and easy to use in states of acute craving, not dependent on technical equipment and therefore readily applicable to everyday life situations.

### Automatic vs. controlled responses

Individuals are often unable to consciously inhibit automatic responses when confronted with desirable food [13–15]. The dual-process model distinguishes between two systems regarding the evaluation of craved stimuli in the information processing domain; the rapidly operating impulsive system, functioning mostly automatic and out of conscious control, and the slower reflective system, operating in a more deliberate and controlled way [7, 16, 17]. An automatic process underlying addiction is the automatic tendency of approaching craved stimuli (i.e. food, alcohol, cigarettes) over neutral ones, known as the approach bias [16, 18]. A link between approach biases and food consumption was demonstrated by a stimulus–response compatibility (SRC) computer task, which showed a stronger approach tendency for food in overeaters than in normal eaters [19]. Another study found that food cravers showed a stronger automatic approach bias towards food than low cravers [20]. A prior trial utilizing the imaginal retraining technique (see section below) for high-calorie foods [21] showed that applying the task was successful in reducing craving, also suggesting a link between approach biases and food craving.

As relapse occurs even though people make the conscious decision to quit and not engage in certain behaviours, it seems to be important to tackle the automatic system when trying to prevent relapses. Research has shown that cognitive biases such as the approach bias can be alleviated through a process called approach bias modification (ABM), which is a form of cognitive bias modification (CBM). In particular, the approach–avoidance task (AAT) has shown promising results in modifying approach biases among many addictive behaviours and substance use disorders, such as drinking [22] and smoking [23]. The instructions of the AAT are explicit in that unhealthy stimuli need to be pushed away as quickly as possible using a joystick, and neutral stimuli are pulled towards oneself. Research has shown that the approach bias in substance use disorders could be reduced with the help of ABM and that relapse rates and craving dropped accordingly [24]. While a meta-analysis uncovered that the efficacy of ABM for eating disorders had fluctuating effect sizes (Hedges’ *g*) across 13 studies between 0.09 and 1.18 [25], one study has shown that after only one training session, the AAT decreased approach tendencies for unhealthy foods in a sample of obese individuals, which was maintained over the following days [5]. A systematic review [26] summarized the effectiveness of approach bias modifications for smoking, alcohol use, and unhealthy eating behaviour across eighteen studies, of which seven focused on food consumption alone. Out of those seven studies, five had successfully retrained the approach bias to unhealthy food. Furthermore, four out of the seven studies found a significant reduction in unhealthy eating behaviour, one of the studies reported both a reduced approach bias and a significant reduction in eating behaviour [26]. However, while ABM studies often show promising results in the laboratory, this is not always the case in clinical settings [26]. This may be because ABM with the original computer-based AAT poses practical limitations, such as the need to perform the task on a computer and the use of repetitive stimuli. Unfortunately, programming an individualized computerized-AAT for each participant would be a time- and cost-consuming endeavour and is thus usually not feasible. In addition, the outcomes of ABM studies are usually assessed immediately rather than over the long term. Moreover, it is not possible to personalize the AAT for each participant to match their specific preferences regarding craved stimuli (i.e. sort of food, brand, way of consumption), which raises questions whether displayed stimuli actually induce craving in the participant and whether the stimuli are representative of individual preferences. Since there is a need for craving reducing interventions which can be applied in moments of acute and high craving, a number of classical ABM techniques are not feasible when specialist equipment, such as computers and joysticks, are required to perform the task.

### Imaginal retraining

Imaginal retraining is a non-computerized technique taking the limitations mentioned before into account, merging imaginal and behavioural components. Previous studies have demonstrated the efficacy of imaginal retraining across a variety of addictive behaviours, such as excessive high-calorie food consumption [21], problem drinking [27] and cigarette smoking [28]. Imaginal retraining transfers the classical AAT into one’s own imagination. Participants are asked to imagine the craved stimuli (i.e. high-calorie food) at their usual place of consumption, followed by a negative mood induction (frowning in disgust and contemplating negative thoughts). Instead of pushing away a joystick as in the classical AAT-version, the imagined craved object needs to be thrown away before the “inner eye” executing a forceful throwing movement physically extending one’s arm.

Imaginal retraining, as the classical AAT, relies on the concept of embodied cognition, namely the notion that there are automatic links between motor movements and perceptions [29] and that cognitions, emotions and movements are interrelated [24, 30], but operationalizes these ideas more consistently.

In day to day life, time-consuming self-help interventions can be hard to implement and maintain. Consequently, a benefit of dismantling effective mechanisms would be to design simpler and briefer interventions. Moreover, previous imaginal retraining interventions were usually implemented for the duration of 1 month [21, 27, 28]. A question of interest is therefore whether a single implementation of the imaginal retraining technique could already have a significant and relevant effect on reducing craving and appraisal for high-calorie food.

### The present study

Since it has not yet been assessed, which of the multiple components of imaginal retraining are responsible for its efficacy, the aim of this study is to uncover the mechanism accountable for craving reduction; in particular, whether the execution of a physical arm movement is necessary. The multiple components identifiable for imaginal retraining are (a) imagining pushing the stimuli away while imagining it becoming smaller, (b) accompanying the imagined pushing with an actual movement, while imagining the stimuli becoming smaller, and (c) shrinking (zooming out) of the desired stimuli, while neither imagining nor performing a movement.

Those three experimental conditions are contrasted against a commonly used approach to curb cravings (d) thought suppression and two control conditions: (e) merely looking at the desired stimuli and (f) a counter-movement condition in which a neutral drink is pulled towards oneself (and pleasurably consumed) in imagination only (for an extensive explanation of the six conditions, see the ‘Experimental conditions’ section).

Out of the total six conditions, a positive effect on craving regarding high-calorie food is hypothesized for the four experimental groups (imaginal retraining with movement, imaginal retraining without movement, zooming, as well as thought suppression), while for the two control conditions (merely taking a look at the picture and the counter movement) no positive effect is expected. It is of interest whether any of those components convey a significant treatment effect in and of itself and whether a one-time implementation of one of the components of the imaginal retraining technique is already effective in significantly reducing craving levels and individual picture appraisal.

## Methods

### Sample

The present study was part of a retrospective follow-up, which succeeded a completed randomized controlled trial assessing the long-term efficacy of imaginal retraining in obese and overweight women (*N* = 384). Initial inclusion criteria were a BMI ≥ 25, age between 18 and 75 years and self-reported excessive consumption of high-calorie foods and the desire to change this behaviour. Exclusion criteria consisted of a history of anorexia, bulimia, psychosis or suicidality.

In the present study, 113 women (29.4% of the baseline sample) completed the additional assessment of the 1-year follow-up study investigating craving effects. Participants who only completed the follow-up assessment and participants who completed both, the follow-up as well as this additional study, did not vary with regards to characteristics such as weight in kg, weight loss since the baseline assessment and trait craving. The participants did not receive a detailed briefing about the aims of the supplementary experiment at hand. Therefore, prevailing expectations regarding the efficacy of anti-craving techniques can be precluded. All participants had received the initial manual with instructions for the imaginal retraining techniques as part of their previous participation upon completion of the post assessment, therefore the retraining concept was not new to most of them. However, it is important to note that not all participants indicated to have read or used the manual in the past year, which demonstrates that those individuals had in fact never used it.

The initial and the follow-up trials were registered with the German Clinical Trials Register (DRKS00016860, DRKS00021044). Ethical approval was obtained from the local psychological ethics committee of psychologists of the University Medical Center Hamburg (Germany; LPEK-0104). This study was registered and conducted simultaneously with another trial dismantling the effects of a single dose imaginal retraining in a sample of smokers [31].

### Invitation and survey

Assessments were implemented online through the platform Questback/UniPark^®^. In consensus with the guidelines of the European General Data Protection Regulation (GDPR), participants IP addresses were not stored. In the baseline assessment, participants were asked to enter an anonymous email address, to which invitations for the newly set-up, voluntary 1-year follow-up assessment was sent. Informed consent was obtained electronically after providing detailed information regarding the content of the follow-up assessment as well as data protection. Next, in the scope of the follow-up study, a questionnaire battery, including psychological scales was administered.

### Questionnaires

Only questionnaires directly relevant to the study are quoted in the following. A variety of sociodemographic and eating-related characteristics including age, weight, height, diet/sport in the last 12 months, instructions followed and manual use in the last 12 months were assessed. Furthermore, the global item of the WHO Quality of Life (WHOQOL-BREF [32], which was used as an index of quality of life, was administered.

### Procedure

After participants completed the online 1-year follow-up assessment, they indicated their current level of craving for high-calorie foods such as sweets and crisps on a visual analogue scale (VAS) from 0 (none) to 100 (very strong) in 10 point intervals. Subsequently, three food-related pictures were shown one by one in fixed order; the first picture displaying a range of healthy foods such as fruits and vegetables, the second displaying a smiling woman biting into a chocolate bar, and the third picture showing a large variety of unhealthy foods such as popcorn, fries, coke, burgers, donuts and crisps. All pictures were rated on a scale from 0 (repulsive) to 100 (very appealing) with a slider (10 point intervals). Picture 1 (variety of healthy foods) acted as a control picture, in which reduction in craving was not expected following the intervention. Participants were then occupied with an irrelevant filler questionnaire, the PHQ-9 [33], to divert their attention away from the pictures they had just been exposed to. Subsequently, a randomized allocation to one of the six interventions (see section ‘Experimental conditions’) took place. Five interventions involved looking at or mentally manipulating a photo displaying a variety of unhealthy sweet- and savoury foods. The photo was standardized across conditions to ensure that the interventions could be compared to one another and thus participants were not instructed to envision an individualized craved object like in the original imaginal retraining task. Only the counter-movement condition received a photo displaying a neutral water bottle, as approaching unhealthy food possibly would have increased craving and was thus deemed unethical. After indicating whether the participants had genuinely performed the intervention (participants were informed that indicating “no” would not have any negative implications), they rated the three pictures (same order) as well as their current level of craving for high-calorie food anew. Their previous responses were not shown. Participants then indicated, whether they felt like they had experienced a real change, no change, whether they only responded in an affirming way because they assumed it was expected of them, or whether they felt like a change occurred for a different reason than the intervention (open text field). At the end of the survey, participants indicated whether they had answered all questions truthfully and were compensated with a manual containing exercises for relaxation and self-compassion.

### Experimental conditions

Participants were randomly assigned to one out of six conditions. In each condition, a picture of a variety of unhealthy high-calorie foods was displayed, following a short instruction:

1. *Control condition:* “Please take a quick look at the picture displaying high-calorie foods. Only press “continue” after you have actually followed the instruction.” (Word count original German text: 19).
2. *Thought suppression:* “Please do the following: Look at the picture, then close your eyes and try not to think about the picture for a few seconds. Please carry out this exercise even if it feels a little strange. Only press “continue” after you have actually followed the instruction.” (Word count original German text: 56).
3. *Zooming:* “Please do the following: Look at the picture. Then close your eyes and imagine the picture slowly getting smaller. Please carry out this exercise even if it feels a little strange. Only press “continue” after you have actually followed the instruction.” (Word count original German text: 53).
4. *Retraining with movement:* “Please do the following: Look at the picture and imagine how you throw away the sweets/crisps in the picture and how in your imagination the picture slowly becomes smaller. Please carry out the actual arm movement, as if you were throwing away the sweets/crisps with contempt. Please carry out this exercise even if this feels a little strange. Only press “continue” after you have actually followed the instruction.” (Word count original German text: 76).
5. *Retraining without movement:* “Please do the following: Look at the picture and imagine throwing away the sweets/crisps in the picture and the picture slowly becoming smaller. Execute the movement in your imagination only. Please carry out this exercise even if it feels a little strange. Then, press “continue”.” (Word count original German text: 60).
6. *Counter-movement:* “Please do the following: Imagine that you are having a delicious drink and that you exaggeratedly set it a bit too high—like in the picture—so that your gaze is directed slightly upwards. Please also carry out this exercise if it feels a little strange. Only press “continue” after you have followed the instructions.” (Word count original German text: 64).

### Statistical analysis

Within-group difference analysis concerning effects on the main outcome variables craving and individual picture appraisal were calculated using paired sample *t*-tests and effect sizes are shown in Cohen’s *d* [34]. In case of non-normal data (Shapiro–Wilk test of craving or picture appraisal scores *p* < 0.05), a non-parametric Wilcoxon paired *t*-test was computed and effect sizes were reported in *r*. As the hypothesis regarding craving reduction was directed, two-tailed *p*-value results were divided by two. For individual picture appraisal, there were no concrete hypotheses, which is why two-tailed *p*-values were reported. Between-group differences regarding the main outcome variables were assessed using one-way ANOVAs and in case of non-normal data by Kruskal–Walis (K-W) tests with effect sizes in *η²*. As picture 1–3 differed in content (healthy vs. non-healthy food), they were analysed individually. Results were first obtained for all participants and in a second step excluding participants indicating not having followed the instructions. An explorative descriptive analysis regarding subjective perception of ‘real change’ was conducted. An additional between-group difference analysis was run with manual usage (yes vs. no) as reported by participants as covariate, i.e. univariate ANCOVA, with group factor on post craving as well as individual picture appraisals, in order to check whether using the manual in the past 12 months had any confounding effect on the outcomes across all groups. Due to the explorative nature of this study, it was not corrected for multiple comparisons. All statistical analyses were conducted with IBM SPSS Statistics 25.

## Results

### Sample characteristics

The sample consisted of 113 women with a mean age of about 50 years (range: 25–72) and a mean BMI of 32.26 (BMI ranged between 21.26 and 56.88). 34.2% of the women showed a BMI between 25 and 29.9 and are therefore classified as overweight and 58.6% had a BMI over 29.9 and are therefore considered obese. Since the baseline assessment, 7.2% of the participants reached a BMI indicating normal weight (between 18.5 and 24.9). As they entered the baseline trial with a BMI ≥ 25 being overweight and reporting excessive consumption of high-calorie foods, they were not excluded from the sample of this study. For group allocation see Table 1. Pre-intervention, participants showed a low to medium intrinsic craving (36 on a scale of 1 to 100) across groups. Pre-intervention the groups differed at trend level regarding picture appraisal rating for picture 2 (woman biting into a chocolate bar) and manual use in the past year (see Table 1). No significant group differences regarding sample characteristics were found between the six conditions.

**Table 1:** Demographic and eating-related characteristics of the sample (*N* = 113).

Groups								
Characteristics	Total	Control (1)	Thought suppression (2)	Zooming (3)	Retraining with movement (4)	Retraining without movement (5)	Counter-movement (6)	Statistic
	(*N* = 113)	(*n* = 18)	(*n* = 21)	(*n* = 24)	(*n* = 18)	(*n* = 17)	(*n* = 15)	(df: 5, 107)
Age in years	49.74 (10.82)	49.72 (10.30)	52.57 (10.17)	48.54 (11.11)	48.67 (12.52)	49.18 (10.64)	49.67 (11.01)	*F* = 0.38, *p* = 0.861
BMI	32.26 (6.89)	31.47 (4.43)	31.59 (7.13)	31.98 (6.50)	32.43 (7.43)	32.68 (6.04)	33.98 (10.01)	*F* = 0.29, *p* = 0.919
Height in cm	165.30 (6.54)	166.22 (7.14)	165.57 (6.80)	165.04 (6.85)	164.56 (6.56)	165.53 (5.92)	164.87 (6.51)	*F* = 0.15, *p* = 0.981
Weight in kg	88.11 (18.82)	87.06 (14.07)	86.88 (21.61)	87.15 (18.81)	87.57 (18.48)	89.39 (15.91)	91.87 (24.68)	*F* = 0.17, *p* = 0.972
Started diet/sport in the past 12 months	33.6%	16.7%	28.6%	25.0%	50.0%	47.1%	40.0%	*F* = 1.45, *p* = 0.213
Instructions not followed	10.6%	22.2%	4.8%	8.3%	5.6%	5.9%	20.0%	*F* = 1.14, *p* = 0.343
Manual use last year	41.6%	33.3%	57.1%	41.7%	55.6%	29.4%	26.7%	*F* = 2.22, *p* = 0.057
Subjective real change post intervention	24.8%	22.2%	23.8%	16.7%	38.9%	35.3%	13.3%	*F* = 0.97, *p* = 0.439
QoL	3.65 (0.83)	3.50 (0.92)	3.57 (0.75)	3.58 (0.78)	4.00 (0.69)	3.71 (0.99)	3.53 (0.92)	*F* = 0.89, *p* = 0.492

*Notes*. BMI = Body-Mass-Index; QoL = WHOQOL-BREF [23] global item score.

### Within-group differences

In three of the six conditions (1. *Control [only looking at picture],* 2. *thought suppression* and 6. *counter movement*) neither a significant reduction in craving nor individual picture appraisal (pictures 1–3) was found (see Table 2). All results remained non-significant after removing participants indicating not having followed the instructions in each condition.

**Table 2:** Pre-post comparisons within and across conditions for craving and pleasantness ratings.

	Craving			Picture 1			Picture 2			Picture 3		
	Pre	Post	Statistics	Pre	Post	Statistics	Pre	Post	Statistics	Pre	Post	Statistics
Control	5.17 (3.05)	4.83 (3.13)	*t*(17) = 1.14, *p* = 0.135*	9.56 (1.79)	9.56 (1.58)	*Z* = −0.21, *p* = 0.832	7.00 (2.91)	6.78 (2.92)	*t*(17) = 0.81, *p* = 0.430	5.56 (2.94)	5.61 (3.07)	*t*(17) = −0.27, *p* = 0.790
Thought suppression	4.38 (2.52)	4.10 (2.05)	*t*(20) = 0.54, *p* = 0.300*	9.62 (2.12)	9.52 (1.81)	*Z* = −0.41, *p* = 0.683	5.43 (2.68)	5.00 (2.67)	*t*(20) = 1.44, *p* = 0.165	3.86 (2.80)	3.33 (2.48)	*Z* = −1.15, *p* = 0.251
Zooming	4.83 (2.82)	4.92 (3.26)	*Z* = −0.26, *p* = 0.397*	9.96 (1.78)	9.42 (2.45)	*Z* = −1.22, *p* = 0.223	7.54 (2.57)	7.17 (2.81)	*t*(23) = 1.04, *p* = 0.309	6.04 (2.99)	4.92 (3.23)	*Z* = −2.07, *p* = 0.038
Retraining with movement	4.00 (2.25)	3.50 (2.26)	*t*(17) = 1.49, *p* = 0.078*	8.89 (2.22)	9.06 (2.07)	*Z* = −1.73, *p* = 0.083	6.28 (2.30)	5.22 (2.39)	*t*(17) = 5.13, *p* *<* 0.001	4.89 (3.09)	3.83 (2.28)	*t*(17) = 2.37, *p* = 0.030
Retraining without movement	4.47 (3.36)	4.18 (3.09)	*Z* = −1.51, *p* = 0.066*	9.82 (1.47)	9.12 (2.06)	*Z* = −1.38, *p* = 0.167	6.00 (3.14)	4.94 (3.03)	*t*(16) = 3.65, *p* = 0.002	3.65 (2.62)	2.94 (2.30)	*Z* = −2.41, *p* = 0.016
Counter-movement	4.73 (2.84)	4.80 (2.37)	*t*(14) = −0.19, *p* = 0.425*	9.47 (2.17)	9.53 (2.10)	*Z* = −0.58, *p* = 0.564	7.73 (2.22)	7.67 (2.38)	*t*(14) = 0.37, *p* = 0.719	5.13 (3.25)	5.07 (3.22)	*t*(14) = 0.56, *p* = 0.582
Total (between-group effect)	4.60 (2.78)	4.40 (2.74)	*X*^2^(5, *N* = 113) = 3.31, *p* *=* 0.326*	9.58 (1.92)	9.37 (2.01)	*X*^2^(5, *N* = 113) = 1.85, *p* *=* 0.869	6.65 (2.73)	6.12 (2.88)	*X*^2^(5, *N* = 113) = 16.13, *p* = 0.006	4.89 (3.01)	4.28 (2.91)	*X*^2^(5, *N* = 113) = 11.98, *p* *=* 0.035

Notes. Descriptive pre and post data include means and standard deviations.

*One-tailed *p*-values are reported.

Due to non-normal data of the difference scores (Shapiro–Wilk test of craving scores for the *zooming condition (group 3) p* < 0.05), non-parametric Wilcoxon paired *t*-tests were computed. Craving was not significantly reduced (*Z* = −0.26, *p*_one-tailed_ = 0.397, *r* = 0.04). Similarly, picture appraisal for picture 1 (fruits and vegetables) and picture 2 (woman biting into a chocolate bar) did not change (see Table 2). However, for picture 3 (variety of unhealthy high-calorie foods) a significant reduction in picture appraisal could be observed (−18.5%; *Z* = −2.07, *p*_two-tailed_ = 0.038, *r* = 0.30).

In the *retraining condition with movement (group 4)*, craving was reduced at a trend level (−12.50%; *t*(17) = 1.49; *p*_one-tailed_ *=* 0.078; *d* = 0.35). While analyses of the picture appraisal did not show a significant reduction for picture 1 (fruits and vegetables; +1.9%; *Z* = −1.73, *p*_two-tailed_ = 0.083, *r* = 0.29), a significant reduction in pleasantness ratings for picture 2 (woman biting into a chocolate bar; –16.9%; *t*(17) = 5.13; *p*_two-tailed_ *<* 0.001 *d* = 1.21) as well as for picture 3 (variety of unhealthy high-calorie foods; –21.7%; *t*(17) = 2.37; *p*_two-tailed_ *=* 0.030; *d* = 0.56) could be observed. However, after the removal of one participant because of the indication not having followed the instructions of the intervention, the effect on picture 3 failed to reach significance and only an effect at trend level could be observed (−20.5%; *t*(16) = 2.06; *p*_two-tailed_ *=* 0.056; *d* = 0.50). In addition, the results in craving reduction did not remain at trend level significance after the removal of the participant (−11.4%; *t*(16) = 1.33; *p*_one-tailed_ *=* 0.102; *d* = 0.32).

In the *retraining condition without movement (group 5)* craving was reduced at a trend level (−6.5%; *Z* = −1.51, *p*_one-tailed_ = 0.066, *r* = 0.26). Individual analyses of the pictures did not show a significant reduction in appraisal for picture 1 (fruits and vegetables; –7.1%; *Z* = −1.38, *p*_two-tailed_ = 0.167, *r* = 0.24), but for picture 2 (woman biting into a chocolate bar; –17.7%; *t*(16) = 3.646; *p*_two-tailed_ *=* 0.002; *d* = 0.88) and for picture 3 (variety of unhealthy high-calorie foods; –19.5%; *Z* = −2.41, *p*_two-tailed_ = 0.016, *r* = 0.41. However, the results for craving reduction did not remain at trend level significance after removing one participant who indicated not having followed the instructions (−6.2%; *Z* = −1.27, *p*_one-tailed_ = 0.103, *r* = 0.22).

For exploratory purposes, an additional post hoc analysis was conducted in which the *retraining condition with movement (group 4)* and the *retraining condition without movement (group 5)* were evaluated together. Due to non-normal data (Shapiro–Wilk test of craving scores for the *retraining condition with and without movement (group 4 and group 5) p* < 0.05), a non-parametric Wilcoxon paired *t*-test was computed. Craving was significantly reduced (−9.5%; *Z* = −1.89, *p*_one-tailed_ = 0.030, *r* = 0.32). Individual picture appraisal for picture 1 (fruits and vegetables; –2.7%; *Z* = −0.61, *p*_two-tailed_ = 0.541, *r* = 0.10) was not reduced, while appraisal for picture 2 (woman biting into a chocolate bar; −17.1%; *t*(34) = 6.09; *p*_two-tailed_ *<* 0.001; *d* = 1.03) and for picture 3 (variety of unhealthy high-calorie foods; –20.8%; %; *Z* = −3.08, *p*_two-tailed_ = 0.002, *r* = 0.52) was reduced significantly.

### Between-group difference analysis

Due to non-normal data (Shapiro–Wilk for craving and all picture appraisals for all groups *p* < 0.05), non-parametric K-W tests were computed. No significant omnibus effect for the group factor concerning craving or picture 1 (variety of healthy foods) could be observed (see Table 2). Post picture appraisal for picture 2 (woman biting into a chocolate bar; *X*^2^(5, *N* = 113) = 16.13, *p* = 0.006) and picture 3 (variety of unhealthy high-calorie foods; *X*^2^(5, *N* = 113) = 11.98, *p* = 0.035); see Table 2) differed significantly across groups with medium effect sizes (*η²* = 0.10 and *η²* = 0.07, respectively). A Bonferroni post hoc pairwise comparison of the groups showed a difference in picture appraisal for picture 2 (woman biting into a chocolate bar) at trend level between group 2 (thought suppression) and group 6 (counter movement; *p*_adjusted_ = 0.086). The post hoc comparison also showed a trend level difference in picture appraisal for picture 3 (variety of unhealthy high-calorie foods) between group 5 (retraining condition without movement) and group 1 (control group; *p*_adjusted_ = 0.086).

### Exploratory descriptive analyses of subjective changes

In total, 24.8% of participants reported to have experienced a real subjective change in craving after their intervention (see Table 1). No significant difference could be found regarding subjective change between groups (*F*(5,107) = 0.971, *p* = 0.439).

### Within-group difference analysis of manual users vs. non-users

ANCOVA analyses were run anew with group factor and manual usage in the past year (yes vs. no) as a covariate. Manual usage during the past year did not account for any significant explained variance in post craving (*F*(1,112) = 0.107, *p* = 0.744), nor individual picture appraisal (picture 1: *F*(1,112) = 1.319, *p* = 0.253); picture 2: (*F*(1,112) = 2.125, *p* = 0.148); picture 3: *F*(1,112) = 0.006, *p* = 0.937) post intervention.

## Discussion

The present study was aimed at dismantling the various components of imaginal retraining to evaluate which ones are most effective in reducing craving for high-calorie foods. We hypothesized, that groups containing the components zooming, imaginal retraining with or without a movement and thought suppression have an advantage over the control condition and the counter movement condition. Indeed, the results of this study showed that the imaginal retraining condition with and without movement prompted reduction in craving at trend level (−12.5% and −6.5%). In the retraining condition including a movement, individual evaluation of pleasantness for both pictures featuring unhealthy foods, showed a significant reduction for picture 2 (−16.9%; woman biting into a chocolate bar) and picture 3 (−21.7%; variety of unhealthy high-calorie foods). The retraining condition without a movement also showed a significant reduction in pleasantness ratings for both pictures displaying unhealthy foods (picture 2, −17.7% and picture 3, −19.5%). In addition, the zooming condition displayed a significant reduction in picture appraisal for picture 3 (−18.5%). None of the participants indicated to have reported a change because it was expected of them.

The results are in line with our hypotheses, showing a higher reduction in craving for both conditions involving imaginal retraining with an imagined or actual movement. As expected, no reductions in either individual picture appraisal or craving could be observed in the control and counter movement condition. In addition, no significant reductions could be found for picture 1, which displayed a variety of healthy foods, speaking for a specific effect of the interventions on craved, high-calorie foods. Although the effects of craving reduction in both imaginal retraining conditions were small (with movement *d* = 0.35, without movement *r* = 0.26) and not robust (trend level significance did not remain after removal of one participant who indicated not having followed the instructions in both imaginal retraining groups), they were observed after a single dose of imaginal retraining only. Moreover, for individual unhealthy picture appraisal reduction the effect sizes in both retraining conditions were small to large (condition with movement *d* = 1.21 and *d* = 0.56; without movement *d* = 0.88 and *r* = 0.24), which indicates that the brief imaginal retraining technique may be particularly effective in reducing appraisal of unhealthy stimuli. As previous studies [26, 28] suggest a dose-effect relationship, a continual application of the exercise might increase its efficacy. The two imaginal retraining conditions are rather similar, because the same sensorimotor units are involved for imagined and actual movements [35]. Because separately the two conditions only showed reductions at a statistical trend level, an exploratory post hoc analysis of both conditions joint was conducted to increase the power of the analysis. A significant reduction in craving (−9.5%) with a medium effect size (*r* = 0.32) and unhealthy picture appraisal for picture 2 (woman biting into a chocolate bar; –17.1%) and picture 3 (variety of unhealthy high-calorie foods; –20.8%) with medium to large effect sizes (*d* = 1.03 and *r* = 0.52, respectively) could be observed.

In sum, the results suggest, that the imagined or actual execution of an arm movement has a benefit over merely zooming an object in imagination alone. Since less cognitive resources are needed to execute the technique upon frequent training, it needs to be assessed whether the actual movement might add on an effect to the retraining with an imagined movement upon progressive practice. For now, we can conclude that there is no apparent difference in efficacy between the condition with or without an actual movement, and hence that the movement possibly might be dispensable. This notion is supported by a recent study [31] on dismantling imaginal retraining components in a sample of smokers. Here, imaginal retraining without an arm movement led to a higher reduction in craving after a single application of the technique. The movement component could act as a distraction from the main task, as it requires additional attention focused on the movement of the arm.

### Limitations

One component that is usually inherent to the imaginal retraining technique is the fact that the user can imagine their individual craved stimuli before their ‘inner eye’. In the scope of this study, for the purpose of better controllability, participants were confronted with preselected stimuli on a computer screen. This poses the limitation that the ‘problem food’ that acts as an object of craving to the participant might not have been displayed on the photo that was shown. As the photo used for the imaginal retraining task contained a range of foods, it might have had the advantage of displaying the craved foods of some individuals, however, it also deviates from the original technique of picturing a particular stimulus in line with one’s personal cravings. It was not assessed, whether participant’s specifically craved objects were represented in this photo. Furthermore, the photo contained a wide variety of foods, arguably difficult to remember and picture before the ‘inner eye’ and possibly too many stimuli to imagine pushing away from oneself. Besides, the variety displayed on the photo contained sweet- and savoury foods, which might not be appetizing to some people and thus might not elicit a high craving effect in itself. For future trials the utilization of a wider range of pictures is desirable.

In conclusion, participants might not have experienced high levels of craving because of the lack of individuality of the food and its portrayal. Furthermore, ratings of craving pre-intervention were only low to medium with a mean of 36 on a scale ranging from 1 to 100. As the imaginal retraining technique is supposed to be helpful to the user in situations of acute craving, future studies should consider inducing high levels of craving first, followed by the utilization of the imaginal retraining techniques. It is important to note that not all components used in the original imaginal retraining technique were examined in the present experiment. The original method also works with the component of mood induction, by imagining either an unpleasant feeling (feeling sick from the consumption of a craved object) or a pleasant one (snuggling a pet) when imagining throwing away/pulling towards oneself a craved vs. neutral stimuli, respectively. Future studies should have a closer look at dismantling those components as well. In addition, as the exploratory post hoc analysis of merging groups 4 and 5 (with and without movement) rendered a statistically significant reduction in craving, future studies should adjust the sample size to increase statistical power to detect effects. Furthermore, upcoming trials should consider a guided laboratory environment using a fully balanced factorial design assessing the main effect of movement. For ethical reasons, such as potentially increasing craving and worsen symptoms, this was not deemed possible within the scope of this online study. It is important to address that the effects would not have survived adjustment for multiple comparisons. Nevertheless, observing trend level reductions in craving after a single-dose of imaginal retraining in this exploratory trial are promising, as it paths the way for future studies. Since the trial was part of a 1-year follow-up of a 6 weeks’ intervention trial, participants (including individuals who had been allocated to the control group), had received a manual explaining the complete imaginal retraining technique in the past year. Therefore, a number of participants were already familiar with the method and had further background information about the mechanisms of the technique. It is possible that the participants might have added other imaginal retraining components to their assigned intervention, because they were already familiar with carrying out the exercise in a certain way. However, we asked whether the current given instructions were followed, which most of the participants (89.0%) confirmed and controlled for variance by manual usage by using it as a covariate.

### Conclusion

While this study suggests that the imaginal retraining technique with and without a movement component might be promising as a brief intervention against cravings, future studies should focus on testing this method when participants are either confronted with or imagine their ‘problem food’ that is most craved to ensure individual craving levels are heightened at the time. Future research should also examine whether repeated application of the dismantled components alters the observed effects. In addition, it is of interest, whether the imaginal retraining technique including a movement might be superior to the imaginal retraining technique without a movement upon repeated practice and subsequent mastery of the technique or vice versa. At the same time the effects of the mood induction component, included in the original version of the imaginal retraining manual should be examined further.

Taking into account the insights of previous imaginal retraining studies [26–28] and the findings of the study at hand, participants should be provided with a detailed description of the technique including background information. Following becoming familiar and practicing the techniques, the participants might then be able to apply them in situations of acute craving. In conclusion, although the effects on craving reduction were small, as we looked at a single dose effect, the preliminary results look promising and should be interpreted as an indicator that even a one off imaginal retraining brief intervention could be an effective and immediate self-help technique in situations of craving.
